# Predictors of Alcohol Use, Alcohol-Related Problems, and Substance Use Following Adolescent Metabolic and Bariatric Surgery

**DOI:** 10.1097/AS9.0000000000000461

**Published:** 2024-07-15

**Authors:** Gretchen E. White, Richard E. Boles, Anita P. Courcoulas, Thomas H. Inge, Susan Z. Yanovski, Todd M. Jenkins, Meg H. Zeller

**Affiliations:** From the * University of Pittsburgh, School of Medicine, Pittsburgh, PA; † University of Colorado Anschutz Medical Campus, Aurora, CO; ‡ Lurie Children’s Hospital of Chicago, Northwestern Feinberg School of Medicine, Chicago, IL; § Division of Digestive Diseases and Nutrition, National Institutes of Health, National Institute of Diabetes and Digestive and Kidney Diseases, Bethesda, MD; ‖ Cincinnati Children’s Hospital Medical Center, Cincinnati, OH; ¶ University of Cincinnati College of Medicine, Cincinnati, OH.

**Keywords:** alcohol, alcohol use disorder, Roux-en-Y gastric bypass, vertical sleeve gastrectomy

## Abstract

**Objective:**

To identify factors associated with incident alcohol consumption, hazardous drinking, alcohol-related problems, and substance use up to 8 years following metabolic and bariatric surgery (MBS) during adolescence.

**Background:**

In this cohort, nearly half of those who underwent MBS as adolescents screened positive for alcohol use disorder, symptoms of alcohol-related harm, or alcohol-related problems within 8 years post-surgery. Moreover, persistent or heavy marijuana use following MBS during adolescence is higher than national data.

**Methods:**

This study includes 217 adolescents (aged 13–19 years) enrolled in a 5-center prospective cohort study who underwent Roux-en-Y gastric bypass or vertical sleeve gastrectomy between 2007 and 2011 and were followed for up to 8 years. Participants self-reported alcohol use via the Alcohol Use Disorders Identification Test and substance use for up to 8 years.

**Results:**

Female sex, pre-surgery lower body mass index, and pre-surgery substance use were independently associated with increased risk of incident post-surgery hazardous drinking. Pre-surgery psychiatric counseling was significantly associated with increased risk for new-onset substance use post-surgery. Starting substance use post-surgery or continuing pre- to post-surgery was independently associated with a higher risk of post-surgery hazardous drinking. Greater percent weight loss, starting post-surgery or continuing pre- to post-surgery psychiatric counseling, using alcohol, and hazardous drinking were independently associated with a higher risk of post-surgery substance use.

**Conclusions:**

Future research with a nonsurgical control group should be examined to further elucidate the relationships between MBS and alcohol and substance use following surgery during adolescence.

## INTRODUCTION

The literature in adult patients undergoing metabolic and bariatric surgery (MBS) indicates that Roux-en-Y gastric bypass (RYGB) and possibly vertical sleeve gastrectomy increase the risk for alcohol-related problems and substance use.^1–4^ An adult study has also shown the prevalence of alcohol use disorder (AUD) and unspecified substance use are higher after MBS compared with patients who had undergone abdominal surgeries.^5^ Data from longitudinal studies indicate that being male, younger age, smoking, lower social support, depression, maladaptive eating (emotional eating), and regular alcohol consumption or pre-surgical AUD predict alcohol-related problems in adults following MBS.^2,3,6^ Similarly, male sex, younger age, low household income, antidepressant use, history of psychiatric hospitalization, smoking, and alcohol consumption predict post-MBS substance use (primarily marijuana).^2^

A series of studies emerging from the Teen-LABS consortium cohort and affiliated TeenView series studies shed initial light on substance use patterns in adolescent patients who underwent MBS.^7–9^ An initial report at 2 years post-MBS identified that being older at the time of surgery and having lower pre- or post-surgery body mass index (BMI) were predictors for increased alcohol use in contrast to a nonsurgical comparator group, which showed age and BMI to be unrelated to alcohol use.^7^ In the longer term, within 8 years post-surgery, nearly half of Teen-LABS participants screened positive for AUD and reported symptoms of alcohol-related harm or alcohol-related problems.^8^ With regard to other substance use, a recent report demonstrated that the prevalence of current (ie, past 30 days) marijuana use 6 years following MBS was similar to both national rates and a nonsurgical comparator group.^9^ However, persistent or heavy marijuana use (daily or near-daily) among adolescents currently using marijuana following MBS was notably high (43%)^9^ compared to national data (23%).^10^ While preliminary, current use (within the past 30 days) of conventional cigarettes appeared to double national rates.^9^ The present study expands upon our prior work^7–9^ by identifying factors associated with incident alcohol consumption, hazardous drinking, alcohol-related problems, and use of a broader range of substances, up to 8 years following MBS during adolescence.

## METHODS

### Data Source

Teen-LABS has previously been described.^8^ Briefly, Teen-LABS^11,12^ is a prospective observational cohort study of 242 adolescents (13–19 years of age) who underwent their first MBS at 1 of 5 clinical centers throughout the United States. Teen-LABS participants were enrolled between 2007 and 2011 after achieving candidacy based on clinical eligibility.^13^ Written informed consent was obtained from participants 18 or 19 years old. For participants younger than 18 years old, caregivers gave written informed consent and participants gave assent. The investigative team informed all participants that responses were confidential. Institutional review boards at each center approved the study protocol. The study is registered at ClinicalTrials.gov (NCT00474318).

Data collection procedures have been described in a previous report on this cohort.^8^ Research staff conducted standardized assessments at the clinical site with participants within 30 days pre-surgery, 6 months post-surgery, and annually thereafter for up to 8 years post-surgery. Assessments were conducted without the caregiver in the room, and all research data, including alcohol and drug measures, were kept confidential and collected independent of clinical care. To decrease participant burden, the year 7 assessment was designed as a brief telephone assessment and, therefore, did not include alcohol-related measures. The clinical teams counseled all participants on alcohol use but did not have access to research assessments disclosing the frequency and quantity of alcohol use. This cohort, including the participant flow diagram, has previously been described.^8^ Briefly, included participants underwent RYGB or vertical sleeve gastrectomy and completed alcohol and drug questionnaires pre-surgery and at least 1 post-surgery assessment.

### Definitions

#### Alcohol and Substance Use

All alcohol-related measures were defined using the Alcohol Use Disorders Identification Test (AUDIT), a validated 10-item test designed to assess alcohol use and the consequences of alcohol use in the prior 12 months.^14^ Teen-LABS participants completed the AUDIT at baseline and annual follow-up visits, with the exception of year 7. Definitions of alcohol use, elevated AUDIT-Consumption (AUDIT-C) score, and substance use in this cohort have been previously described.^8^ An elevated AUDIT-C^15^ score (ie, the threshold used to identify hazardous drinking) is ≥2 in adolescents <18 years old,^16^ ≥3 in females ≥18 years old, and ≥4 in males ≥18 years old.^17^ An elevated AUDIT^15^ score (ie, the threshold used to identify alcohol-related problems or harmful drinking) is ≥2 in adolescents <18 years old^16^ and ≥8 in adults ≥18 years old.^17^ Elevated AUDIT and AUDIT-C scores were calculated according to participant age at each assessment. Following surgery, participants were asked whether the effect of alcohol changed since surgery using the question, “Does the effect of alcohol on you differ from before surgery?” Substance use included any self-reported use of opiates, amphetamines, hallucinogens, inhalants, marijuana, cocaine, and phencyclidine “other than as prescribed by a physician” in the past 12 months (yes/no).

#### Incidence of Alcohol and Drug-Related Outcomes

When each alcohol and drug-related outcome (ie, alcohol use, hazardous drinking, alcohol-related problems or harmful drinking, and substance use) was absent at baseline but present at follow-up, that outcome was defined as incident (or post-MBS onset) alcohol use, hazardous drinking, alcohol-related problems or harmful drinking, and substance use, respectively.

#### Pre- and Post-Surgery Predictor Measures

Participant and caregiver sociodemographic characteristics were self-reported pre-surgery. BMI (kg/m²) was calculated from participant weight measurement to the nearest and height measurement to the nearest inch during a local laboratory visit.^18^ Mental health was measured using the norm-based component scores from the Medical Outcomes Study 36-item short-form health survey where a higher score indicated better functioning.^19^ Binge eating disorder and loss of control eating were derived from the Questionnaire of Eating and Weight-Patterns–Revised^20^ and have previously been described.^21^ Past-year psychiatric medication use, past-year psychiatric counseling, lifetime history of psychiatric hospitalization, and current cigarette use were measured with Teen-LABS forms.^22^ Past-year psychiatric counseling excluded pre-surgical psychiatric assessment by the MBS clinical team.

### Statistical Analysis

We conducted analyses using SAS version 9.4 (SAS Institute, Cary, NC). *P* values are 2-sided, and *P*<0.05 indicates statistical significance. We did not adjust for multiple comparisons.^23^ Among participants with pre-surgery alcohol use, we assessed the proportion who felt that the effect of alcohol differed after surgery using descriptive statistics. All longitudinal analyses used a person-level random intercept and controlled for age as a fixed effect to account for the transition to being able to legally consume alcohol in the United States and clinical site as a fixed effect because it was related to missing follow-up data. Assessment-level data were set to missing when participants reported being pregnant.

Methods describing the modeling of Pre-Surgery Predictors of Incident Post-Surgery Alcohol and Substance Use and Pre-to-Post-Surgery Changes Associated With Post-Surgery Alcohol and Substance Use are given in the Supplemental Materials http://links.lww.com/AOSO/A376.

## RESULTS

Pre-surgery characteristics of this cohort and the flow of participants from recruitment to analysis sample have been previously described.^8^ Among eligible participants with pre-surgery alcohol data, post-MBS data were obtained from 88%, 88%, 86%, 82%, 83%, 86%, 87%, and 80% at 6 months and 1, 2, 3, 4, 5, 6, and 8 years, respectively. Briefly, most participants were female (76%), White (72%), and non-Hispanic (93%) and had undergone RYGB (71%; Table 1). The median (25th–75th percentile) pre-surgery age was 17 (15–18) years, and the median BMI was 51 (45–59) kg/m^2^. Eight percent of participants drank alcohol within the 12 months pre-surgery, 3% had hazardous drinking, 5% had alcohol-related problems, and 4% used substances. Of the 8% (n=18) of participants with pre-surgery alcohol use, the proportion who agreed that the effect of alcohol differed from before surgery ranged from 40% 2 years following surgery to 77% 6 years following surgery (66.7%, 40.0%, 50.0%, 64.3%, 58.3%, 76.9%, and 60.0% at 1, 2, 3, 4, 5, 6, and 8 years post-surgery, respectively). The incidence of alcohol use in the 8 years following MBS in this population has been previously described.^8^

**TABLE 1.** Pre-Surgery Demographic and Clinical Characteristics of Analysis Sample (n=217)

	n	%*
Female	164	75.6
Age, y; median, 25th–75th percentile	17	15–18
Race		
White	157	72.4
Black	48	22.1
Other	12	5.5
Hispanic ethnicity	16	7.4
Caregiver relationship status		
Never married	28	13.3
Divorced, separated, or widowed	54	25.6
Married or living as married	129	61.1
Caregiver education		
≤High school	86	40.8
Some college	85	40.3
≥College degree	40	19.0
Caregiver household income (U.S. $)		
<25,000	78	37.5
25,000–74,999	81	38.9
≥75,000	49	23.6
Surgical procedure		
Roux-en-Y gastric bypass	155	71.4
Vertical sleeve gastrectomy	62	28.6
Body mass index, median (25th–75th percentile)	51.0	45.4–58.9
SF-36 mental component summary score, median (25th–75th percentile)	51.9	43.4–56.8
Psychiatric medication	62	28.7
Psychiatric counseling	63	24.5
Lifetime history of psychiatric hospitalization	21	9.7
Binge eating disorder	28	12.9
Loss of control eating	62	28.6
Frequency of alcohol use		
Never	199	91.9
Monthly or less	14	6.5
2–4 times/month	4	1.8
≥2 times/week	0	0.0
Average quantity of drinks per drinking day		
0	199	92.1
1–2	10	4.6
3–4	3	1.4
≥5	4	1.8
AUDIT-C score, median (25th–75th percentile)	0	0–0
Hazardous drinking†	7	3.2
AUDIT score, median (25th–75th percentile)	0	0–0
Alcohol-related problems‡	11	5.1
Symptoms of alcohol dependence§	3	1.4
Alcohol-related harm‖	7	3.2
Substance use	8	3.7
Cigarette smoking	3	1.4

* Unless otherwise specified.

† AUDIT-C score ≥2 for participants <18, ≥3 for females ≥18, and ≥4 for males ≥18.

‡ AUDIT score ≥2 for participants <18, ≥8 for participants ≥18.

§ Not being able to stop drinking once started, failing to meet normal expectations because of drinking, or needing a drink in the morning to get going in the past 12 months.

‖ Feeling guilt or remorse, being unable to remember, injuring someone, or eliciting concern due to drinking in the past 12 months.

AUDIT indicates Alcohol Use Disorder Identification Test; AUDIT-C, Alcohol Use Disorder Identification Test-Consumption; and SF-36, 36-item short-form health survey.

Reproduced with permission from White et al.^8^

The prevalence of any substance use was 3.7%, 7.3%, 15.6%, 19.0%, 20.0%, 16.9%, 24.3%, and 27.5%, at pre-surgery, 1, 2, 3, 4, 5, 6, and 8 years post-surgery, respectively. Substance use was primarily marijuana use. Among participants with any substance use, the proportion reporting using marijuana, with or without the addition of other substances, ranged from 94% to 100% at each timepoint. Opiates were used by 10%–22%, cocaine by 3%–18%, hallucinogens by up to 15%, and amphetamines by up to 11% of participants who used substances in years 1–7 post-MBS. Inhalants and PCP were rarely used. The proportion of participants with substance use who reported using marijuana only was 88%, 86%, 79%, 76%, 78%, 74%, 68%, and 70% at 1, 2, 3, 4, 5, 6, and 8 years post-surgery, respectively.

### Pre-Surgery Predictors of Incident Post-Surgery Alcohol and Substance Use

Unadjusted pre-surgery predictors of incident post-surgery alcohol use, hazardous drinking, alcohol-related problems, and substance use are given in Supplemental Table 1 http://links.lww.com/AOSO/A376. In adjusted models, pre-surgery older age, history of psychiatric hospitalization, and substance use were significantly associated with increased risk of incident post-surgery alcohol use, while female sex, lower BMI, and substance use were independently associated with increased risk of incident post-surgery hazardous drinking (Table 2). Psychiatric counseling was significantly associated with increased risk for new-onset alcohol–related problems and substance use post-surgery. History of psychiatric hospitalization was also significantly associated with a higher risk for new-onset alcohol–related problems (Table 2).

**TABLE 2.** Adjusted Pre-Surgery Predictors of Incident Post-Surgery Alcohol and Substance Use

	Alcohol use		Hazardous drinking*		Alcohol-related problems†		Substance use	
	aHR (95% CI)	*P*	aHR (95% CI)	*P*	aHR (95% CI)	*P*	aHR (95% CI)	*P*
Male sex (vs female)	1.12 (0.76–1.65)	0.58	0.33 (0.17–0.67)	0.002	1.31 (0.68–2.52)	0.41	1.11 (0.64–1.91)	0.71
Age, per 1 year older	1.24 (1.11–1.37)	<.001	1.08 (0.94–1.23)	0.29	0.94 (0.79–1.10)	0.43	0.99 (0.86–1.13)	0.88
Body mass index, per 5 kg/m^2^ lower	1.09 (0.99–1.20)	0.08	1.18 (1.02–1.37)	0.03				
Psychiatric counseling (vs no)					1.85 (1.03–3.33)	0.04	1.74 (1.06–2.84)	0.03
History of psychiatric hospitalization (vs no)	1.71 (0.97–3.01)	0.06			2.57 (1.17–5.66)	0.02		
Substance use (vs no)	6.14 (1.43–26.28)	0.01	4.39 (1.29–15.00)	0.02			NA	

Cox proportional hazards models adjusted for other variables as indicated in this table, as well as a clinical site.

* AUDIT-C score ≥2 for participants <18, ≥3 for females ≥18, and ≥4 for males ≥18.

† AUDIT score ≥2 for participants <18, ≥8 for participants ≥18.

aHR indicates adjusted hazard ratio; AUDIT, Alcohol Use Disorder Identification Test; AUDIT-C, Alcohol Use Disorder Identification Test-Consumption; CI, confidence interval; and NA, not applicable.

### Pre- to Post-Surgery Changes Associated With Post-Surgery Alcohol and Substance Use

Unadjusted associations of pre-and post-surgery participant characteristics with post-surgery alcohol use, hazardous drinking, alcohol-related problems, and substance use are given in Supplemental Table 2 http://links.lww.com/AOSO/A376. In adjusted models, pre-surgery history of psychiatric hospitalization, starting substance use post-surgery or continuing pre- to post-surgery, stopping substance use from pre- to post-surgery, and post-surgery cigarette use were independently associated with a higher risk of post-surgery alcohol use (Table 3). Starting substance use post-surgery or continuing pre- to post-surgery was independently associated with a higher risk of post-surgery hazardous drinking. Starting substance use post-surgery or continuing pre- to post-surgery was associated with a higher risk of post-surgery alcohol–related problems. Greater percent weight loss, starting psychiatric counseling post-surgery or continuing pre- to post-surgery, starting alcohol use post-surgery or continuing pre- to post-surgery, and starting hazardous drinking post-surgery or continuing pre- to post-surgery were independently associated with a higher risk of post-surgery substance use. Adjustments for the calendar year of data collection had a negligible impact on parameter estimates.

**TABLE 3.** Adjusted Associations of Pre- and Post-Surgery Participant Characteristics With Post-Surgery Alcohol and Substance Use, Among Participants Without the Respective Condition in the Year Before Surgery

	Alcohol use		Hazardous drinking*		Alcohol-related problems†		Substance use	
	aRR (95% CI)	*P*	aRR (95% CI)	*P*	aRR (95% CI)	*P*	aRR (95% CI)	*P*
Pre- to post-surgery changes								
Percent weight loss, per 5% more weight loss							1.07 (1.01–1.14)	0.03
SF-36 mental component summary score, per 10 lower							1.10 (1.00–1.23)	0.06
Pre- and post-surgery status								
Psychiatric counseling								<.001
Started vs never							2.13 (1.40–3.23)	
Stopped vs continued							0.64 (0.46–0.89)	
Continued vs never							2.45 (1.54–3.90)	
Lifetime history of psychiatric hospitalization		0.03		0.05				
Post-surgery vs never	1.23 (0.88–1.71)		0.79 (0.51–1.24)					
Pre-surgery or pre- and post-surgery vs never	1.51 (1.04–2.19)		1.34 (0.74–2.43)					
Alcohol use								0.01
Started using vs never							2.11 (1.33–3.34)	
Stopped using vs continued							1.17 (0.34–3.99)	
Continued using vs never							3.54 (1.28–9.75)	
Elevated AUDIT-C score								0.05
Started/continued vs never							1.48 (1.08–2.02)	
Stopped vs started/continued							0.73 (0.11–4.93)	
Substance use		<.001		<.001		<.001		
Started/continued vs never	1.47 (1.26–1.73)		2.12 (1.53–2.93)		2.13 (1.56–2.93)			
Stopped vs started/continued	2.86 (2.06–3.97)		1.90 (0.76–4.73)		1.74 (0.77–3.92)			
Post-surgery cigarette use	1.34 (1.14–1.57)	<.001	1.42 (0.97–2.09)	0.07			1.40 (0.98–1.99)	0.06

Poisson mixed models with robust error variance adjusted for sex, age, clinical site, and significant baseline variables are shown in Table 1 and other variables as indicated in this table.

* AUDIT-C score ≥2 for participants <18, ≥3 for females ≥18, and ≥4 for males ≥18.

† AUDIT score ≥2 for participants <18, ≥8 for participants ≥18.

aRR indicates adjusted relative risk; AUDIT, Alcohol Use Disorder Identification Test; AUDIT-C, Alcohol Use Disorder Identification Test-Consumption; CI, confidence interval; NA, not applicable; and SF-36, 36-item short-form health survey.

## DISCUSSION

In this prospective cohort study of individuals who underwent MBS during adolescence, we identified pre-surgery factors associated with incident post-surgery alcohol use, hazardous drinking, alcohol-related problems, and substance use and pre- to post-surgery changes associated with the same post-surgery substance use measures. To our knowledge, this is the first study to assess predictors of alcohol use, hazardous drinking (elevated AUDIT-C score), and alcohol-related problems (elevated AUDIT score) following MBS during adolescence into young adulthood. Initial comparisons showed that adolescents who completed MBS were similar to youth with severe obesity 2 years after surgery regarding rates of alcohol use and lower than national rates of alcohol use during the same period though concerning subgroup signals of alcohol consumption were reported.^7^

Consistent with the broader developmental literature,^24,25^ our results illustrate common pathways between substance use and alcohol use post-surgery since initiating alcohol and substance use were associated with increased risk of the other. Interestingly, stopping versus starting or continuing substance use post-surgery was also associated with an increased risk of alcohol use. Furthermore, post-surgery cigarette use was associated with increased risk of post-surgery alcohol use. Alcohol use, substance use, and cigarette smoking are strongly associated within the general US population of adolescents and young adults.^26^ Adolescents and young adults who use multiple substances (ie, alcohol, cigarettes, and substances) have a higher risk of substance use disorders and physical injuries in later adulthood.^26–29^

Our findings suggest that lower pre-surgery BMI and higher pre- to post-surgery percent weight loss are associated with increased risk of hazardous drinking and substance use, respectively. We did not find any association between BMI and weight loss with alcohol-related problems perhaps because the AUDIT also captures alcohol dependence and alcohol-related harm, while the AUDIT-C only measures hazardous drinking.^30^ Findings from our study add to those from a recent publication involving our present cohort that found that 6 years post-MBS during adolescence, current marijuana use was not significantly associated with BMI or percent weight loss.^9^ The differences between the 2 studies may be at least partially explained by the current study controlling for post-surgery cigarette use, using different measurement approaches (ie, yes/no vs the number of occasions and past 12 months vs past 30 days) and not limiting substances to marijuana use alone. While the overwhelming majority of people in this study who used substances used marijuana, nearly 20% used opiates or cocaine. Our findings are also contrary to results from Reslan et al^31^ in which adult RYGB patients with lower percent weight loss had a higher likelihood of endorsing substance misuse. Using similar measures as those used in our study, results from the Longitudinal Assessment of Bariatric Surgery-2 cohort suggest no association between percent weight loss and substance use following bariatric surgery.^2^ More research should be done to understand the associations between BMI, weight loss, and drug and alcohol use in this population.

Age was not associated with post-surgery hazardous drinking, alcohol-related problems, or substance use. However, older age was associated with initiation of alcohol use post-surgery, which may reflect the developmental transition seen in the general US population since the average age of first drinking alcohol is 15 years old.^32^ We also found that male sex was protective against incident post-surgery hazardous drinking, which is consistent with evidence from the general US population that shows that the prevalence of AUD is higher in adolescent females than in adolescent males.^33^ In adults aged 18 and older, the prevalence of AUD is higher in males than in females.^33^ Our findings that there is no association between sex and consuming alcohol are also consistent with those from the general US population that shows that male and female adolescents have a similar prevalence of alcohol use.^34^ While age and sex were not predictive of alcohol use problems and substance use in contrast to adults after MBS,^2^ other risk factors do appear to overlap among adolescents and adults, including smoking and history of psychiatric hospitalization/counseling.^2,3,6^

Strengths of this study are its large sample, longitudinal design, high retention, and use of multiple validated and reliable alcohol use measures. Limitations include a primarily White and female sample, reducing generalizability. Alcohol and substance use were self-reported and subject to underreporting, particularly in an adolescent population when alcohol use is not legal.^35^ This may make comparisons to the adult literature on alcohol use following MBS difficult because there may be less underreporting in the adult population. Moreover, our survey of substance use behaviors was single-item past-year endorsements, leaving chronicity and severity of use and risk factors for such use unknown. Sample size and low prevalence of some predictors (ie, pre-surgical cigarette smoking and receiving substance use treatment) limited our ability to test some factors previously shown to be associated with problematic alcohol use and substance use in adults post-MBS. Furthermore, Teen-LABS only collected data on whether the effect of alcohol changed after MBS and not how it changed. Possible changes in the effect of alcohol include becoming intoxicated on a smaller quantity of alcohol than before surgery or experiencing physiological effects of alcohol such as flushing or gastrointestinal distress. Importantly, this paper lacks a nonsurgical control group with obesity, the inclusion of which would allow a more direct examination of substance use risks related to undergoing MBS within a developmental context.

Future research should examine additional predictors of alcohol use, hazardous drinking, and alcohol-related problems and include a more comprehensive qualitative examination of substance use behaviors and their progression to abuse and substance use disorders to develop additional hypotheses. For example, additional factors known to be associated with substance use in adolescents and young adults include alternate tobacco product use, family history of alcohol and drug abuse, stressful life events, parental involvement, parental divorce, peer use, psychopathology, and psychological dysregulation.^36–39^

In this prospective cohort of patients undergoing MBS during adolescence, we identified several nonsurgical risk factors for post-surgery alcohol use, hazardous drinking, and substance use. Routine screening for alcohol problems and substance use should be conducted in clinical settings as adolescent patients age into adulthood. Future research with a nonsurgical control group will further elucidate the relationships between MBS and substance use following surgery during adolescence.

## Supplementary Material


