# Supplementary material for: Predictors of Alcohol Use, Alcohol-Related Problems, and Substance Use Following Adolescent Metabolic and Bariatric Surgery
Source: Ann Surg Open. 2024 Jul 15;5(3):e461. doi: 10.1097/AS9.0000000000000461 (PMC11415110; doi:10.1097/AS9.0000000000000461)
Supplement: Supplementary file 1 [file as9-5-e461-s001.pdf]

Supplemental Materials

Methods

Pre-Surgery Predictors of Incident Post-Surgery Alcohol and Substance Use

Among participants without pre-surgery alcohol use, hazardous drinking, alcohol-related problems/harmful drinking, or substance use, Cox proportional-hazard models were used to identify pre-surgery factors associated with risk of incident post-surgery alcohol use, hazardous drinking, alcohol-related problems, and substance use. Independent variables included age, sex, parental education level, caregiver relationship status, annual household income, surgical procedure (RYGB vs VSG), BMI, SF-36 mental component summary score, psychiatric medication use, and psychiatric counseling in the past 12 months, history of hospitalization for any psychiatric/emotional problems, binge eating disorder, and loss of control eating, and alcohol use, hazardous drinking, alcohol-related problems, and substance use, when applicable. Factors with $P<0.20$ in independent models were entered into a single multivariable model with clinical site, age, and sex (which were forced into the models) and retained via backward elimination if $P<0.10$.

Pre-to-Post-Surgery Changes Associated with Post-Surgery Alcohol and Substance Use

Poisson mixed models with robust error variance were used to identify pre- to post-surgery changes associated with post-surgery alcohol use, hazardous drinking, alcohol-related problems, and substance use, respectively. Pre- to post-surgery changes with $P<0.20$ in independent models were entered into a single multivariable model with clinical site, age, sex, and factors identified as significant in Cox proportional-hazard models and retained via backward elimination if $P<0.10$. The following pre- to post-surgery changes were considered: percent weight loss, change from pre-surgery in SF-36 mental component summary score with

control for pre-surgery values, and pre- and post-surgery psychiatric medication use, psychiatric counseling in the past 12-months, history of hospitalization for any psychiatric/emotional problems, binge eating disorder, loss of control eating, and alcohol use, hazardous drinking, alcohol-related problems, and substance use in models in which they were not the outcome. Post-surgery cigarette smoking was also considered as an independent variable without consideration for pre-surgery smoking because cigarette smoking pre-surgery was too rare. Change in caregiver relationship status, income, and education were not assessed as independent variables. Sensitivity analyses were also performed to examine the potential impact of secular trends by including calendar year as a covariate in all multivariable models.

Supplemental Table 1. Unadjusted associations between pre-surgery characteristics and incident post-surgery alcohol and substance use

	Alcohol use		Hazardous drinking[a]		Alcohol-related problems[b]		Substance use	
	HR (95% CI)	P	HR (95% CI)	P	HR (95% CI)	P	HR (95% CI)	P
Male sex (vs. female)	1.15 (0.79-1.67)	0.46	0.37 (0.19-0.71)	0.003	1.12 (0.59-2.12)	0.72	0.99 (0.58-1.70)	0.98
Age, per 1 year older	1.21 (1.10-1.34)	<.001	1.03 (0.91-1.18)	0.62	0.95 (0.81-1.12)	0.53	1.00 (0.87-1.15)	0.99
Caregiver education								0.26
≤ High school	1.0 (reference)	0.76	1.0 (reference)	0.24	1.0 (reference)	0.81	1.0 (reference)	
Some college	0.89 (0.62-1.27)		1.30 (0.78-2.17)		1.20 (0.64-2.27)		1.53 (0.91-2.55)	
≥ College degree	1.02 (0.64-1.63)		1.66 (0.92-3.01)		1.23 (0.57-2.69)		1.14 (0.58-2.25)	
Caregiver relationship status		0.12		0.75		0.28		0.11
Never married	0.81 (0.47-1.40)		0.98 (0.48-2.01)		1.48 (0.65-3.38)		0.94 (0.46-1.93)	
Divorced, separated, or widowed	1.36 (0.95-1.96)		1.21 (0.72-2.02)		1.64 (0.87-3.08)		1.07 (0.63-1.82)	
Married or living as married	1.0 (reference)		1.0 (reference)		1.0 (reference)		1.0 (reference)	
Caregiver household income (U.S. $)								0.33
≤ 25,000	1.0 (reference)	0.32	1.0 (reference)	0.17	1.0 (reference)	0.78	1.0 (reference)	
25,000-74,999	1.01 (0.70-1.45)		1.65 (0.98-2.78)		0.92 (0.50-1.72)		0.87 (0.53-1.49)	
≥75,000	0.72 (0.45-1.15)		1.32 (0.71-2.48)		0.76 (0.34-1.70)		1.04 (0.56-1.94)	
RYGB (vs. VSG)	0.90 (0.60-1.35)	0.60	0.76 (0.43-1.35)	0.35	0.88 (0.45-1.73)	0.71	0.93 (0.53-1.63)	0.81
Body mass index, per 5 kg/m^2 lower	1.06 (0.97-1.16)	0.19	1.22 (1.06-1.42)	0.01	1.00 (0.85-1.16)	0.95	1.00 (0.89-1.13)	0.98
SF-36 mental component summary score, per 10 lower	1.09 (0.93-1.27)	0.30	1.12 (0.92-1.36)	0.26	1.12 (0.87-1.45)	0.39	1.11 (0.91-1.36)	0.32
Psychiatric medication use (vs. no)	1.29 (0.90-1.83)	0.17	1.36 (0.86-2.17)	0.19	1.51 (0.85-2.70)	0.16	1.46 (0.89-2.40)	0.13
Psychiatric counseling (vs. no)	1.40 (0.98-2.00)	0.07	1.51 (0.95-2.40)	0.08	1.97 (1.11-3.47)	0.02	1.71 (1.05-2.77)	0.03
History of psychiatric hospitalization (vs. no)	1.80 (1.04-3.12	0.04	1.71 (0.92-3.18)	0.09	2.81 (1.31-6.05)	0.01	1.95 (1.02-3.74)	0.04
Binge eating disorder (vs. no)	1.17 (0.75-1.84)	0.49	1.37 (0.76-2.48)	0.30	1.21 (0.58-2.52)	0.61	0.86 (0.45-1.66)	0.66
Loss of control eating (vs. no)	1.02-0.72-1.45)	0.90	1.16 (0.72-1.86)	0.54	1.35 (0.77-2.39)	0.30	1.25 (0.77-2.02)	0.37
Substance use (vs. no)	4.46 (1.05-18.97)	0.04	3.80 (1.17-12.35)	0.03	1.89 (0.46-7.82)	0.38	NA	
Alcohol use (vs. no)	NA		1.58 (0.69-3.66)	0.28	1.84 (0.57-5.95)	0.31	1.63 (0.70-3.78)	0.25
Elevated AUDIT-C score (vs. no)	NA		NA		NA		1.03 (0.14-7.49)	0.98
Elevated AUDIT score (vs. no)	NA		NA		NA		1.91 (0.69-5.26)	0.21

AUDIT, Alcohol Use Disorder Identification Test; AUDIT-C, Alcohol Use Disorder Identification Test-Consumption; CI, confidence interval; HR, hazard ratio; NA, not applicable.

[a] AUDIT-C score ≥2 for participants <18, ≥3 for females ≥18, and ≥4 for males ≥18.

[b] AUDIT score ≥2 for participants <18, ≥8 for participants ≥18.

Supplemental Table 2. Unadjusted associations of pre- and post-surgery participant characteristics with post-surgery alcohol and substance use, among participants without the respective condition in the year before surgery

	Alcohol use		Hazardous drinking[a]		Alcohol-related problems[b]		Substance use	
	RR (95% CI)	P	RR (95% CI)	P	RR (95% CI)	P	RR (95% CI)	P
Pre- to post-surgery change								
Percent weight loss, per 5% more weight loss	1.02 (0.99-1.06)	0.03	1.03 (0.97-1.10)	0.31	1.02 (0.93-1.11)	0.13	1.07 (1.00-1.14)	0.05
SF-36 mental component summary score, per 10 lower	0.96 (0.90-1.02)	0.17	1.08 (0.97-1.20)	0.18	1.39 (1.21-1.60)	0.004	1.18 (1.07-1.30)	<.001
Pre- and post-surgery status								
Psychiatric medication		0.17		0.27		0.02		0.002
Started vs never	1.01 (0.83-1.23)		1.46 (0.97-2.19)		2.04 (1.23-3.37)		1.57 (1.10-2.25)	
Stopped vs continued	0.81 (0.63-1.05)		1.05 (0.66-1.66)		0.77 (0.41-1.42)		0.58 (0.39-0.87)	
Continued vs never	1.28 (1.01-1.61)		1.30 (0.79-2.14)		2.08 (1.06-4.08)		2.08 (1.23-3.53)	
Psychiatric counseling		0.25		0.14		0.004		<.001
Started vs never	1.04 (0.83-1.29)		0.74 (0.50-1.09)		1.70 (0.90-3.22)		2.17 (1.46-3.21)	
Stopped vs continued	0.94 (0.72-1.23)		0.97 (0.58-1.61)		0.47 (0.25-0.89)		0.65 (0.46-0.92)	
Continued vs never	1.28 (0.97-1.67)		1.43 (0.82-2.50)		2.96 (1.60-5.48)		2.85 (1.74-4.68)	
Lifetime history of psychiatric hospitalization		0.003		0.005		0.01		0.001
Post-surgery vs never	1.00 (0.71-1.39)		0.69 (0.44-1.08)		0.52 (0.30-0.88)		0.51 (0.34-0.76)	
Pre-surgery or pre- and post-surgery vs never	1.44 (0.98-2.12)		1.46 (0.78-2.76)		1.20 (0.53-2.72)		0.96 (0.50-1.88)	
Binge eating disorder		0.39		0.43		0.87		0.41
Started/continued vs never	0.75 (0.40-1.37)		1.73 (0.71-4.23)		1.04 (0.27-3.99)		0.61 (0.29-1.27)	
Stopped vs started/continued	1.50 (0.80-2.84)		0.67 (0.23-1.96)		1.19 (0.26-5.32)		1.27 (0.68-2.47)	
Loss of control eating		0.17		0.88		0.03		0.52
Started vs never	0.87 (0.69-1.11)		1.10 (0.67-1.81)		1.12 (0.42-3.03)		1.28 (0.79-2.07)	
Stopped vs continued	0.80 (0.59-1.08)		1.02 (0.63-1.64)		0.70 (0.39-1.25)		0.98 (0.59-1.62)	
Continued vs never	1.32 (0.98-1.77)		1.17 (0.69-1.97)		2.76 (1.40-5.47)		1.38 (0.78-2.43)	
Alcohol use	NA		NA		NA			<.001
Started vs never							2.42 (1.58-3.70)	
Stopped vs continued							1.19 (0.32-4.37)	
Continued vs never							3.31 (1.21-9.05)	
Elevated AUDIT-C score	NA		NA		NA			0.002
Started/continued vs never							1.80 (1.31-2.48)	
Stopped vs started/continued							1.10 (0.18-6.62)	
Substance use		<.001		<.001		<.001	NA	
Started/continued vs never	1.47 (1.24-1.73)		2.11 (1.53-2.91)		3.40 (2.14-5.40)			
Stopped vs started/continued	2.23 (1.67-2.98)		1.75 (0.75-4.06)		1.73 (0.21-14.10)			
Post-surgery cigarette use	1.40 (1.19-1.64)	<.001	1.54 (1.08-2.18)	0.02	1.99 (1.28-3.08)	0.002	1.80 (1.33-2.44)	<.001

AUDIT, Alcohol Use Disorder Identification Test; AUDIT-C, Alcohol Use Disorder Identification Test-Consumption; CI, confidence interval; RR, relative risk. NA, not applicable.

[a] AUDIT-C score ≥2 for participants <18, ≥3 for females ≥18, and ≥4 for males ≥18.

[b] AUDIT score ≥2 for participants <18, ≥8 for participants ≥18.
